# Baseline Raman Spectral Fingerprints of Zebrafish Embryos and Larvae

**DOI:** 10.3390/bios14110538

**Published:** 2024-11-06

**Authors:** Isabel Oliveira Abreu, Cláudia Teixeira, Rui Vilarinho, A. Cristina S. Rocha, Joaquim Agostinho Moreira, Luís Oliva-Teles, Laura Guimarães, António Paulo Carvalho

**Affiliations:** 1 CIIMAR—Interdisciplinary Centre of Marine and Environmental Research, University of Porto, Terminal de Cruzeiros do Porto de Leixões, Av. General Norton de Matos, s/n, 4450-208 Matosinhos, Portugal; aabreu@ciimar.up.pt (I.O.A.); claudia.teixeira@ciimar.up.pt (C.T.); loteles@fc.up.pt (L.O.-T.); 2 Department of Biology, Faculty of Sciences of the University of Porto, Rua do Campo Alegre, s/n, 4169-007 Porto, Portugal; 3 ICBAS—School of Medicine and Biomedical Sciences, University of Porto, Rua de Jorge Viterbo Ferreira, 228, 4050-313 Porto, Portugal; 4 IFIMUP—Institute of Physics for Advanced Materials, Nanotechnology and Photonics, Faculty of Sciences of the University of Porto, Rua do Campo Alegre, s/n, 4169-007 Porto, Portugal; rvsilva@fc.up.pt (R.V.); jamoreir@fc.up.pt (J.A.M.); 5 MARE—Marine and Environmental Sciences Centre/ARNET—Aquatic Research Network, Department of Life Sciences, University of Coimbra, Calçada Martim de Freitas, 3000-456 Coimbra, Portugal; acsrocha@uc.pt

**Keywords:** zebrafish organs, Raman bands, developmental dynamics, amino acids, lipids

## Abstract

As a highly sensitive vibrational technique, Raman spectroscopy (RS) can provide valuable chemical and molecular data useful to characterise animal cell types, tissues and organs. As a label-free, rapid detection method, RS has been considered a valuable asset in forensics, biology and medicine. The technique has been applied to zebrafish for various purposes, including physiological, biochemical or bioaccumulation analyses. The available data point out its potential for the early diagnosis of detrimental effects elicited by toxicant exposure. Nevertheless, no baseline spectra are available for zebrafish embryos and larvae that could allow for suitable planning of toxicological assessments, comparison with toxicant-elicited spectra or mechanistic understanding of biochemical and physiological responses to the exposures. With this in mind, this work carried out a baseline characterisation of Raman spectra of zebrafish embryos and larvae throughout early development. Raman spectra were recorded from the iris, forebrain, melanocytes, heart, muscle and swim bladder between 24 and 168 h post-fertilisation. A chemometrics approach, based on partial least-squares discriminant analysis (PLS-DA), was used to obtain a Raman characterisation of each tissue or organ. In total, 117 Raman bands were identified, of which 24 were well represented and, thus, retained in the data analysed. Only three bands were found to be common to all organs and tissues. The PLS-DA provided a tentative Raman spectral fingerprint typical of each tissue or organ, reflecting the ongoing developmental dynamics. The bands showed frequencies previously assigned to collagen, cholesterol, various essential amino acids, carbohydrates and nucleic acids.

## 1. Introduction

Raman spectroscopy (RS) is an inelastic light scattering technique. It is based on capturing the vibration of molecular bonds elicited by an incident electromagnetic monochromatic light beam, ranging from near-infrared to visible or ultraviolet (UV). Upon incidence with the light beam, the kinetic energy of the incidental particles is not conserved, resulting in losses and gains of energy [1,2]. This energy shift is indicative of discrete vibrational modes of polarisable molecules. It provides qualitative and quantitative assessments of the chemical composition, as well as the molecular structure, of the sample analysed, i.e., molecular fingerprints or fingerprint signatures [3,4]. For this, it has been successfully used in mineralogy and the material sciences. In the last decades, it has been increasingly applied to the investigation of biological samples and their biochemical composition, as well as the diagnosis of disease. It has been used in investigations in pharmacology, cell biology microbiology and toxicology [3,4,5,6,7]. It has also been employed in the diagnosis and prognosis of cancer and other diseases [1,8,9,10,11] and the environmental diagnosis of water quality [2,12].

The Raman spectra obtained from biological samples usually comprise a high number of bands, strongly overlapped. Each band represents a molecular vibration, typically observed within the 300 to 3500 cm^−1^ wavenumber range. These are assigned, among others, to bond vibrations of carbohydrates (470 to 1200 cm^−1^), DNA phosphate groups (980, 1080 and 1240 cm^−1^) and proteins (1500 to 1700 cm^−1^) [3,6]. Other relevant bands are found at wavenumbers ranging from 2700 to 3500 cm^−1^, assigned to vibrations of bonds involving CH, NH and OH stretching vibrations in lipids and proteins [3,6]. Besides the high sensitivity and ability for biochemical characterisation, the technique is reagent-free, requiring minimal or no sample preparation. This is particularly relevant in ecotoxicology, as external perturbations related to sample preparation and analysis may easily result in artefacts. Such artefacts may complicate the assessments to be performed and mislead the inferences drawn from the results obtained. Assessment of tissues as near as possible to physiological conditions (apart from the changes potentially elicited by the chemicals under investigation), is thus better to detect and describe the effects of specific substances, such as chemical toxicants. Additionally, interference of the water present in biological samples can be minimised, allowing for a lower signal-to-noise ratio during the acquisition of Raman spectra [1,12]. Recently, it has also been employed in investigations with zebrafish, as a model for some human diseases, and to investigate chemical bioaccumulation [13,14,15,16].

Zebrafish (*Danio rerio*) is a well-established model organism, commonly used in various research fields, such as developmental biology, pharmacology, veterinary sciences, evolutionary biology, nanotechnology, medicine and in ecotoxicology [11,17,18,19,20]. As such, it has also been used in RS studies, with different aims: (i) assessing the composition of contaminants, their distribution and bioaccumulation; (ii) investigating various biological processes; and (iii) determining the effects of some contaminants. There are several examples of studies focusing on distribution and bioaccumulation of contaminants within embryos, larvae or adult zebrafish [21,22,23,24,25,26]. Other authors have conducted investigations of the zebrafish bone development [13,27], adipogenesis [28], biochemical alterations in the brain or the fatty-acid metabolism [29,30], as well as lipid accumulation and distribution [16]. Ecotoxicological investigation using RS in zebrafish has been carried out, for instance to assess the toxicity of carbon nanotubes [31]. Overall, these works point out the value of applying RS to the investigation and diagnosis of detrimental effects of environmental contaminants on zebrafish. Most of the available studies have, however, been conducted in late larval stages or adult animals. Only a few employed embryos or early larvae, or followed the early developmental windows of high gene transcription related to intense growth and morphogenesis [32]. No RS characterisation of the developmental dynamics in different organs and tissues is available. This is necessary information, as such early life stages in fish development, particularly in zebrafish, are known to be highly sensitive to chemical contaminants, with neuroendocrine receptors already in place and measurable detoxification and biotransformation responses indicative of exposure [17,18,19]. Moreover, during this period, before the yolk sac is completely reabsorbed (or the starting of exogenous feeding), zebrafish can be used as a suitable and sensitive alternative to animal testing under the 3R ethics and the Act for the Welfare of Laboratory Animals. The aim of this study was, therefore, to provide a baseline (fingerprint) characterisation of tissues and organs of zebrafish throughout early development that may be useful to identify hallmark bands and molecules, and plan experiments to investigate relevance for human and environmental health.

## 2. Materials and Methods

### 2.1. Zebrafish Husbandry and Test Organisms

Laboratory-reared adult zebrafish (*Danio rerio*, wild-type AB) were kept in 14 h light: 10 h darkness, at a temperature of 28 ± 1 °C, and pH 7.0. The animals were fed a commercial diet (Zebrafeed by Sparos, Sparos Lda., Olhão, Portugal) twice a day, supplemented with artemia nauplii (*Artemia* spp.) once a week. The day before spawning, a breeding tank was placed inside the main aquarium, with marbles in it to stimulate spawning. Embryos were collected and placed in a Petri dish with water, and fertilised eggs were selected for the experiments. The fertilised embryos were kept in an incubator at 28 ± 0.1 °C with regulated photoperiod, until acquisition of Raman spectra, which was performed on six animals (i.e., three embryos/larvae from two different clutches) from 24 to 168 h post-fertilisation (hpf). The organs, tissues and cells assessed were the iris (24–168 hpf), forebrain (in-between the eyes, 24–168 hpf), melanocytes (48–168 hpf), heart (48–168 hpf), muscle (48–168 hpf) and swim bladder (96–168 hpf). These were selected based on preliminary trials and available information on their response to emerging contaminants of concern [18,19], to be easily accessible and measurable for the purpose of future environmental diagnosis.

### 2.2. Raman Spectroscopy

Acquisition of Raman spectra was performed using an InViaTM Qontor^®^ confocal Raman spectrometer (Renishaw, Kingswood, UK) assembled with a Leica DM2700 microscope (Ernst Leitz GmbH, Wetzlar, Germany). A Cobolt 04-01 Series SambaTM (Hübner Photonics, Kassel, Germany) incident laser was employed. The wavelength of the excitation laser used was 532 nm with a 5× objective. To improve the signal/noise ratio, the acquisition time was 10 s with 4 accumulations; the laser power was set to 5 mW in order to avoid sample heating. Raman spectra were acquired from each investigated tissue/organ in the 100 to 4000 cm^−1^ spectral range, using WiRETM 5.2. (Renishaw Inc., Wotton-under-Edge, UK). Tissue and organ regions were determined under the microscope. Before measurement, embryos were anesthetised with 0.12% tricaine (MS 222). At the end of the acquisitions, the embryos and larvae were alive and apparently in good condition. The Raman spectra were then deconvoluted by fitting a sum of damped oscillator functions and a basis function to simulate the background, using an Igor ProTM (Wavemetrics Inc., Portland, OR, USA, 1998) routine. For the fitting procedure, the area (A), width (W), and frequency (F) of each band were determined and taken as dependent variables (Yi) [12].

### 2.3. Data Analysis

Overall, 4873 spectra were acquired and deconvoluted. From these, 117 Raman bands were identified. Among the identified bands, 24 were found to be well represented in the target organs, allowing for a reliable characterisation of their profile. The criterion to retain bands for data analysis was the presence of the band in at least a time window and organ in >60% of the animals analysed. A partial least squares regression discriminant analysis (PLS-DA) was then carried out for each tissue/organ. In this PLS-1 analysis, the developmental stages (as measured in hpf) were taken as Y variables. The Raman variables derived with the deconvolute fitting (i.e., frequency, width and peak area) were the characterising regressors or descriptors (i.e., X variables). The number of dimensions retained was decided through the comparison of the R^2^ (variance explained) and Q^2^ (estimate of the predictive ability of the model as calculated via cross-validation). The interpretation of the results was performed through the calculation of the scalar projections as described previously [33,34]. Briefly, the scalar projections are an integrated measure of the relationship (covariation) between the descriptors and the developmental stages (hpf). They are obtained by calculating the scalar projections of the developmental stages (Yi) over the descriptor loadings (Xi) in the Cartesian hyperspace formed by the significant PLS components. The scalar projections represent an integrated measure of the relationship (covariance) between the descriptors (Raman variables) and the developmental stages. In addition, the corresponding length of the sum of the scalar projections, herein referred to as vector module, was calculated. The vector module shows high correlation with the variable importance in the PLS and reflects the discrimination of the dependent variables by the descriptors (i.e., Raman variables). The results provided an overall characterisation of each organ and identification of its typical bands. The statistical analysis was performed in Statsoft StatisticaTM 64 (Statsoft Software Inc., Tulsa, UK, 2014).

## 3. Results and Discussion

**Figure 1:**
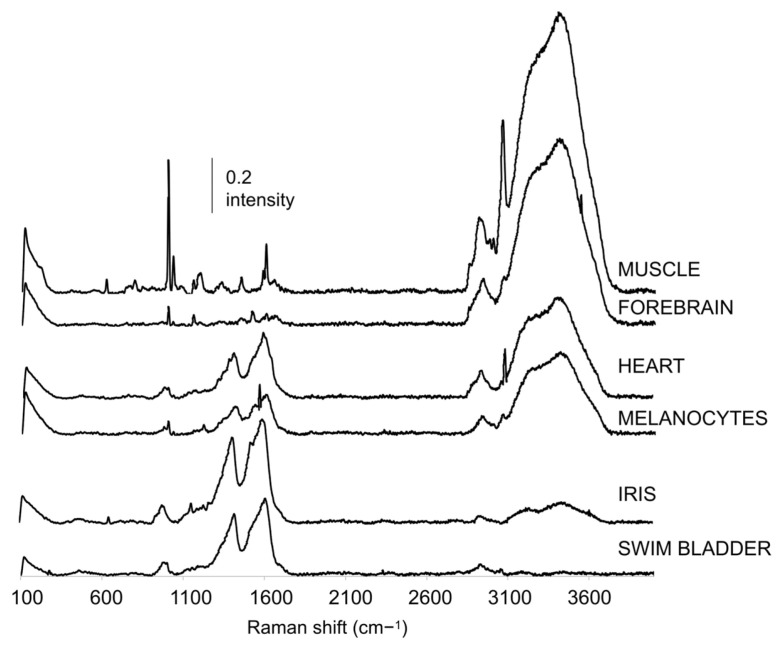
Raman spectra recorded in the 100 to 4000 cm^−1^ spectral range from zebrafish larvae at 144 hpf. A global summary of all bands found for each tissue and organ throughout development (24 to 168 hpf) is presented in Figure 2.

**Figure 2:**
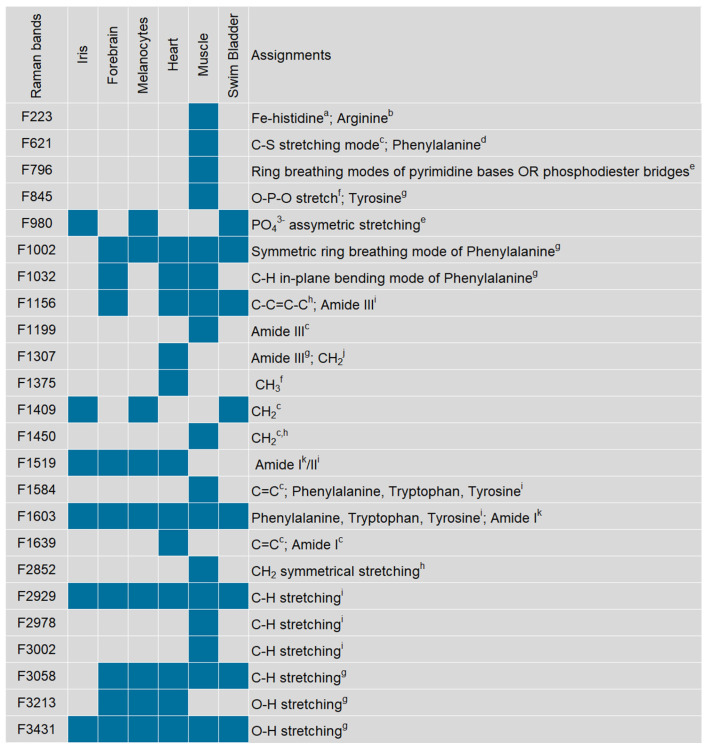
Frequency (F) of Raman bands identified in the spectra recorded from zebrafish embryos and larvae (24 to 168 hpf) in the spectral range 223 to 3431 cm^−1^, and corresponding molecular vibration assignment following the literature. ^a^ [43], ^b^ [44], ^c^ [29], ^d^ [45], ^e^ [14], ^f^ [23], ^g^ [36], ^h^ [15], ^i^ [39], ^j^ [46], ^k^ [47].

The Raman data obtained were analysed to produce a baseline profile throughout early development of the main zebrafish organs and tissues easy to measure during this period. The 24 bands identified and the respective organ/tissues from which they were recorded are shown in Figure 1 and Figure 2. The bands found were in the 223 to 3431 cm^−1^ spectral range.

The molecular vibration assignments found in the zebrafish literature for these bands are presented in Figure 2. Most of them were assigned to vibrations of amino acids and other relevant macromolecules, such as nucleic acids, proteins and lipids [6,35]. Each organ/tissue exhibited a specific Raman spectrum, composed of variables derived from a different number of bands. In particular, the Raman spectra recorded from the iris and the swim bladder were deconvoluted into six and eight bands, respectively; those of the melanocytes and the forebrain comprised nine bands; the heart and muscle Raman spectra were composed of 12 and 17 bands, respectively. Moreover, some bands were found in one organ or tissue only, namely the bands 1307, 1375 and 1639 cm^−1^ in the heart spectra and bands 223, 621, 796, 845, 1199, 1450, 1584, 2852, 2978, and 3002 cm^−1^ in the muscle spectra. Two other bands were exclusively found in the Raman spectra of three organs/tissues: the bands at 980 and 1409 cm^−1^ that could only be observed from the iris, melanocytes and swim bladder (Figure 1 and Figure 2). On the other hand, three bands at 1603, 2929 and 3431 cm^−1^ were found in all organs/tissues, suggesting their possible relation to common structural cell/tissue components. Several of the bands detected were found to be associated with CH vibrations, namely bands 1307, 1375, 1409, 1450, 2852, 2929, 2978, 3002 and 3058 cm^−1^. Stretching vibrations of CH bonds are usually found at higher wavenumbers (2800 to 3100 cm^−1^) [36,37], while bending or scissoring vibration modes are often found at lower frequencies in the spectra (1440 to 1650 cm^−1^) [38]. In zebrafish, bands 1307, 1375, 1409 and 1450 cm^−1^ have been mainly associated with CH bending or scissoring in the eye and heart of larvae [39], as well as the brain [23,29], gonads [29] and liver of adults [36,40,41]. Bands 2852, 2929, 2978, 3002 and 3058 cm^−1^ were found to be related to CH stretching in lipids and proteins of the heart [39], liver [30] and yolk sac [42] of zebrafish embryos and larvae. The band at 2929 cm^−1^, in particular, has been detected previously by other authors in various organs, as either CH_2_ stretching in the somites, yolk sac, vasculature, outer surface and head of zebrafish larvae [15], or CH_3_ stretching in the muscle of larvae [42] and liver of adults [36]. Overall, this provided already a clear distinction among organs, with potential interest for assessment in pollutant-exposure investigations. Specifically, follow-up of such bands upon chemical exposure can bring knowledge on how such treatments can interfere with these bands/molecules (e.g., shifts, intensity differences) and their usefulness to diagnose exposure and characterise modes of action.

**Figure 3:**
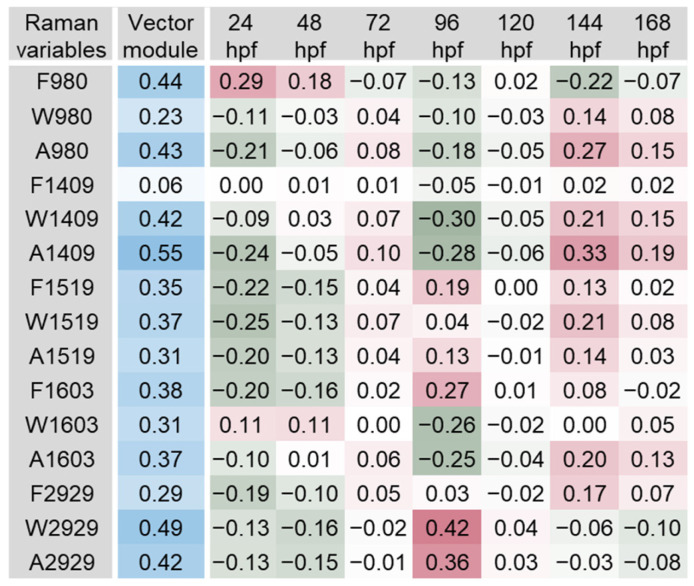
Scalar projections and vector module calculated from the significant components extracted by the partial least squares discriminant analysis (PLS-DA) performed on the iris Raman data. Higher module values (blue shades) indicate a higher discrimination of the developmental time windows. Positive scalar projections (red shades) indicate higher values of the variables, while negative scalar projections (green shades) indicate lower (below the average) values of the variables.

**Figure 4:**
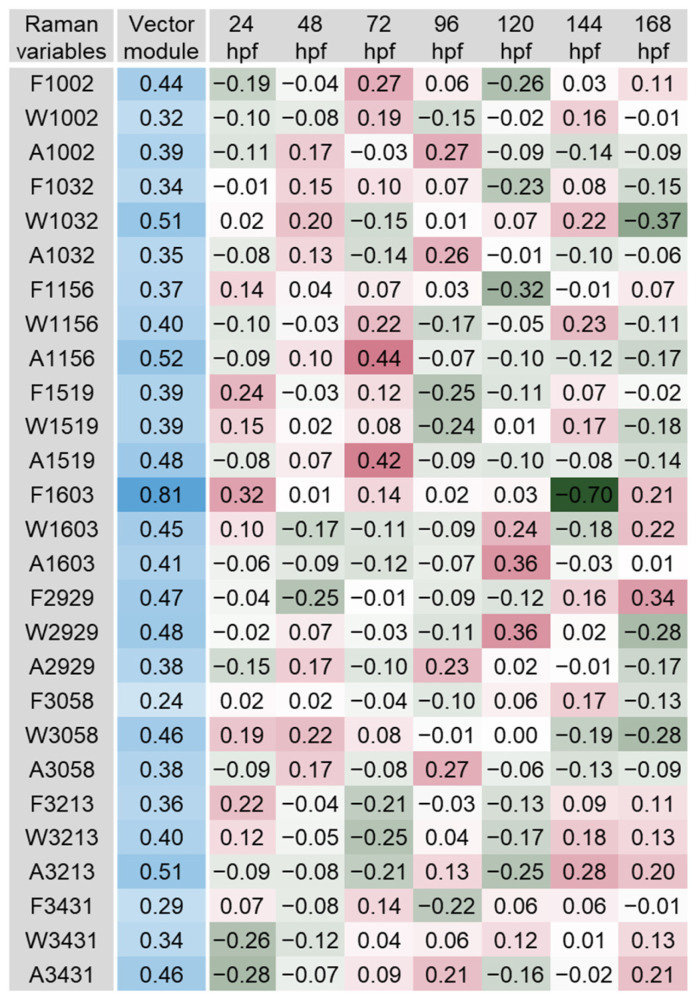
Scalar projections and vector module calculated from the significant components extracted by the partial least squares discriminant analysis (PLS-DA) performed on the forebrain Raman data. Colour legend as in Figure 3.

**Figure 5:**
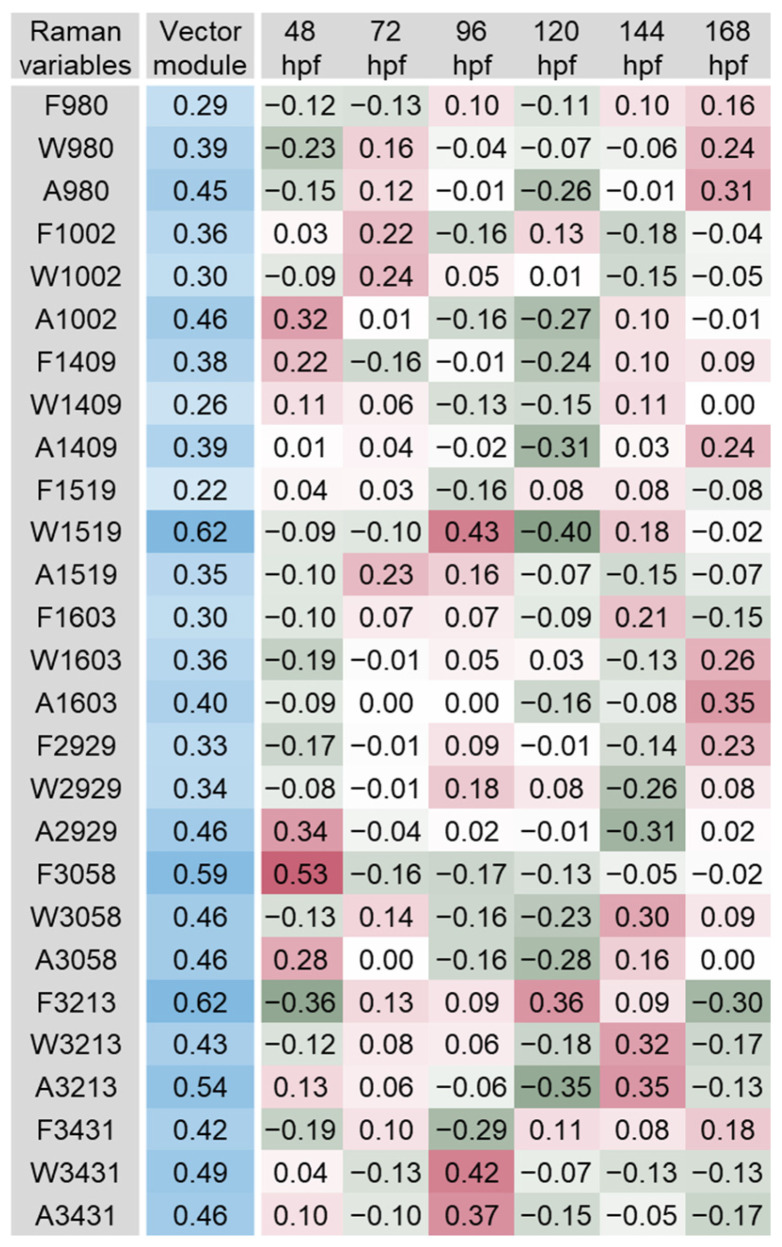
Scalar projections and vector module calculated from the significant components extracted by the partial least squares discriminant analysis (PLS-DA) performed on the melanocyte Raman data. Colour legend as in Figure 3.

**Figure 6:**
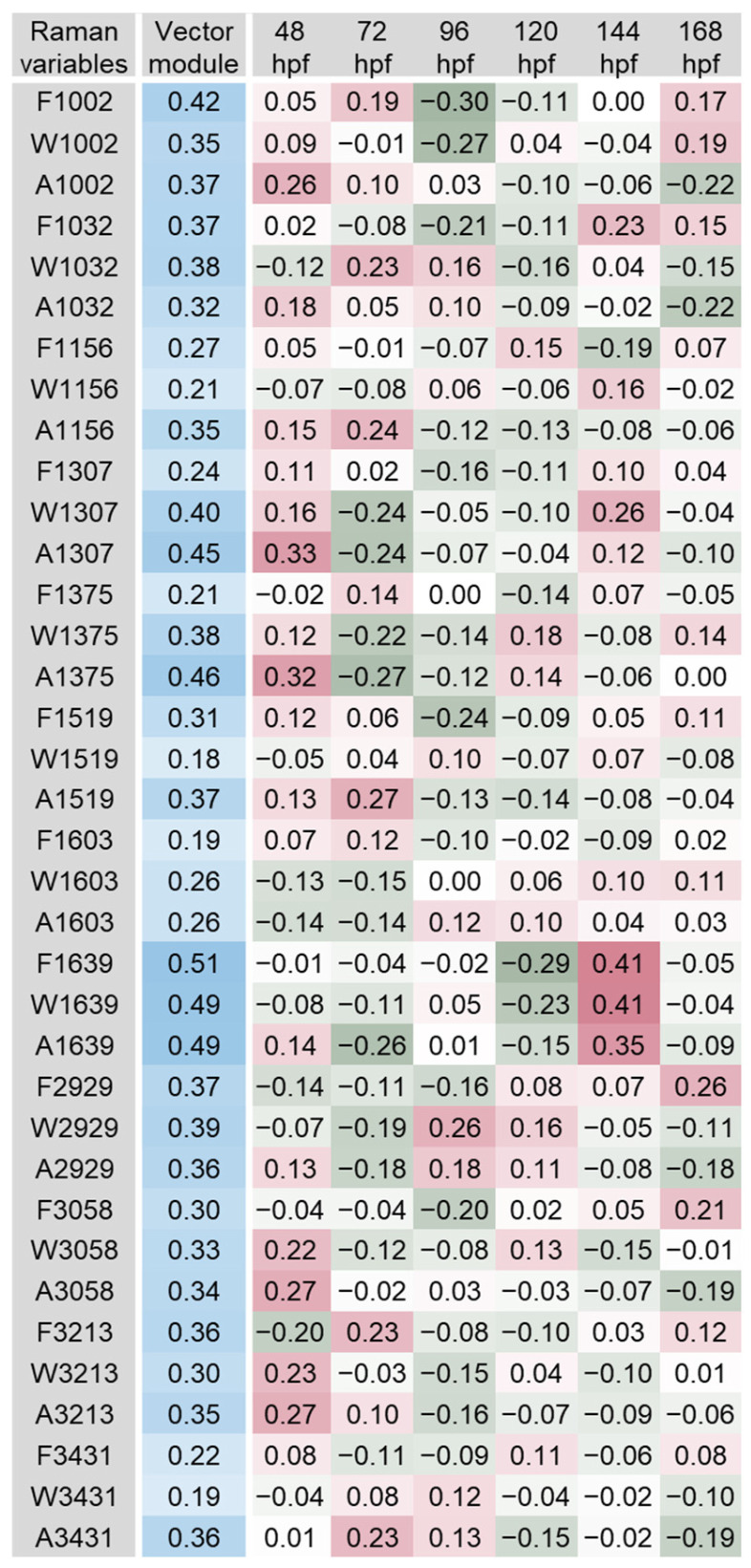
Scalar projections and vector module calculated from the significant components extracted by the partial least squares discriminant analysis (PLS-DA) performed on the heart Raman data. Colour legend as in Figure 3.

**Figure 7:**
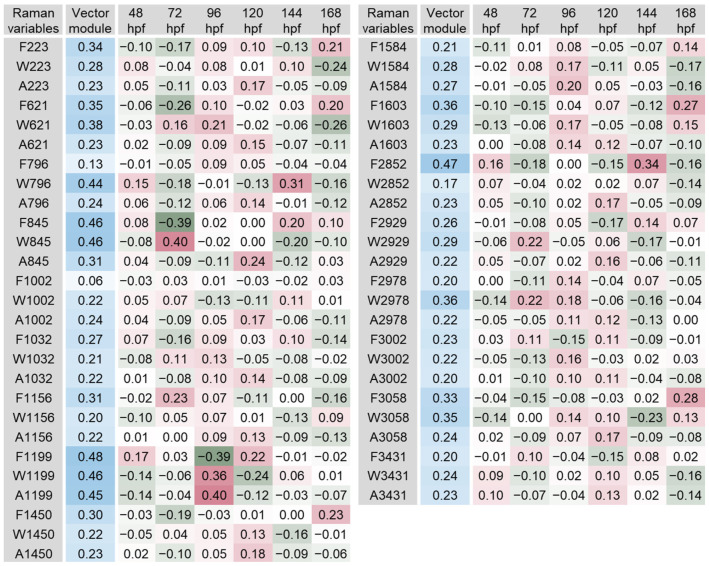
Scalar projections and vector module calculated from the significant components extracted by the partial least squares discriminant analysis (PLS-DA) performed on the muscle Raman data. Colour legend as in Figure 3.

**Figure 8:**
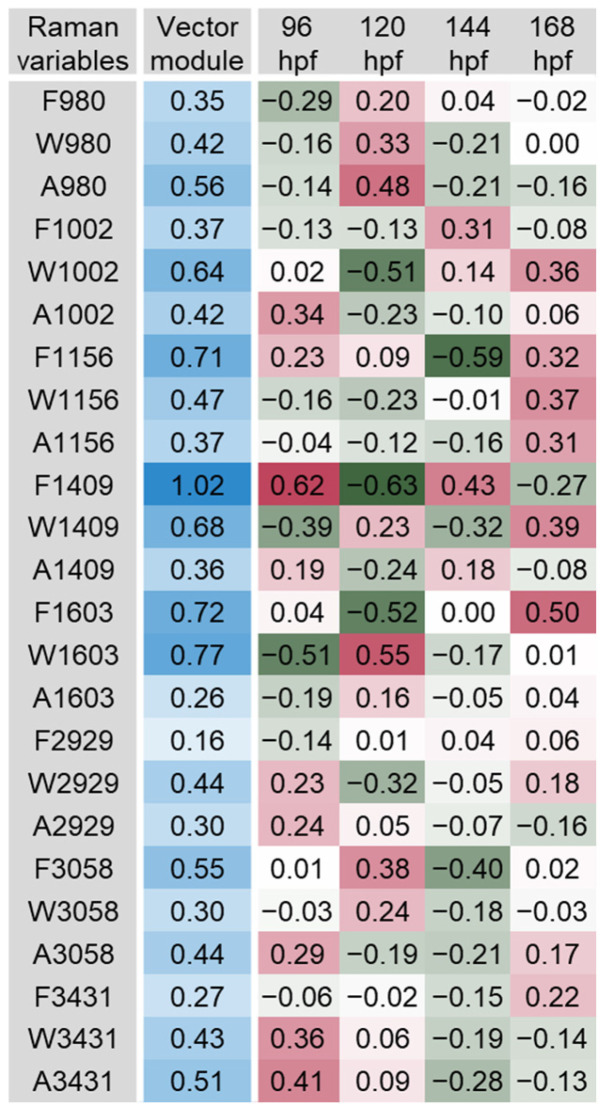
Scalar projections and vector module calculated from the significant components extracted by the partial least squares discriminant analysis (PLS-DA) performed on the swim bladder Raman data. Colour legend as in Figure 3.

The scalar projections, calculated from the significant PLS-DA components extracted, provided an indicative integrated Raman spectral profile for each organ (Figure 3, Figure 4, Figure 5, Figure 6, Figure 7 and Figure 8). The vector module reflected the global discrimination of the developmental time windows by the descriptors—the higher the value, the stronger the discrimination.

Based on the analysis of the vector module, the most relevant variables in the iris spectra were A1409, W2929, F980, A980, A2929 and W1409, with module values between 0.42 and 0.55 (Figure 3). Variable F980 tended to exhibit higher values during early embryonic development (24 and 48 hpf) and take average or lower values after hatching. Frequency band variations due to different laser excitation wavelengths should be related to resonance phenomena [1,12]. However, the differences in the frequency of a band recorded at fixed laser excitation wavelength are usually related to the presence of different molecular conformations and/or intermolecular interactions. Variables A980 and A1409 showed similar patterns over development, which were inverse to that of F980. Smaller values (band area) were observed during embryonic development (especially at 24 hpf) and larger areas at 144 and 168 hpf. The band area is related to the concentration of the molecules assigned, which is lower for smaller areas. Interestingly, the data indicate the values obtained for the relevant variables clearly distinguish 96 hpf from the remaining developmental stages. The distinct values found may be related to optokinetic responses, which can be consistently recorded at 96 hpf [48,49]. These are eye reflexes concomitant with the visual system development in zebrafish. They are triggered by objects moving across the visual field. Moving objects evoke stereotyped tracking eye movements with two components [48]. One is a smooth movement pursuing the moving object; the other is a sudden and rapid (saccadic) movement to reset the eyes after the moving object has left the visual field. This is the first behaviour in the animal requiring the vision of forms and is amenable to measurement with adequate tests [48,49] to better understand its potential relationship to the RS bands obtained. The patterns observed for A2929 and W2929 were also similar, with a trend towards higher values immediately after hatching (96 hpf) and lower or average values before and after this time window. The band width reflects the local environment of the target molecule present in the tissue or organ, with higher values indicating higher disorder and intermolecular interactions. As discussed above, bands at 1409 and 2929 cm^−1^ are likely assigned to vibration modes of CH bonds, i.e., bending/scissoring and stretching, respectively. Band 980 cm^−1^, as well as bands in nearby wavenumbers, have been associated with the symmetric stretching of phosphate (PO_4_^3−^): e.g., amorphous calcium phosphate in developing bones (950 cm^−1^) and apatite in mature bones (960 cm^−1^) in the caudal fin of zebrafish juveniles [14,27] and adults [50]. In this work, band 980 cm^−1^ and 1409 cm^−1^ were detected in the iris, melanocytes (i.e., in highly pigmented organs) and the swim bladder. Further studies envisaging peak assignments in these organs will help clarify which PO_4_^3−^-rich molecules and CH bonds can be detected and their function. Surface-enhanced Raman scattering (SERS) could also help elucidate this matter. As a highly sensitive RS method, SERS uses nanostructured particles and surfaces made of silver or gold to create intense electromagnetic fields, amplifying the Raman signal [51]. In addition, SERS is highly sensitive in the study of small molecules containing nitrogen and oxygen [52], a useful characteristic since some of these pigments can contain nitrogen in their composition [53,54]. Thus, although not suitable for expeditious environmental diagnosis, SERS shows great potential to help clarify the presence of particular molecules within specific organs and the physiological functions in which they may be involved.

In the profile obtained for the forebrain, the most relevant variables were F1603, A1156, W1032 and A3213, with vector module values between 0.51 and 0.81 (Figure 4). Most of those variables showed clear oscillation of their values over time that might be related to the protein turnover involved in developmental processes. Bands 1032, 1156 and 1603 cm^−1^ have previously been associated with amino acids phenylalanine, tryptophan and tyrosine, as well as with amide molecules (e.g., amide III, band 1307 cm^−1^; amide I, band 1603 cm^−1^; Figure 2) in the brain and other organs of zebrafish embryos, larvae or adults [23,29,36,47,55]. These amino acids play a key role in protein synthesis and are precursors of vital neurotransmitters: serotonin (tryptophan), dopamine and epinephrine (phenylalanine and tyrosine) [56]. Amide bonds between two amino acids are called peptide bonds, originating dipeptides. Because the dipeptides formed have an amine group on one extremity and a carboxylic acid on the other, additional amino acids can be covalently bonded to both ends, the vibration modes depending on the backbone molecular structure of these biomolecules [38]. The spectral range 1142 to 1175 cm^−1^ has also been associated with a carotenoid-rich region in the vasculature and outer layer of zebrafish larvae [15,47]. Stretching O-H vibrations indicated by bands 3213 and 3431 cm^−1^ have been related to water and changes in its molecular structure [36]. The four bands identified and the variables extracted from them came out as good candidates for follow-up in experimental studies.

In melanocytes, the most relevant Raman variables were W1519, F3213, F3058 and A3213, with vector module values ranging from 0.54 to 0.62 (Figure 5). Band 1519 cm^−1^ has been associated with carotenoids in the yolk sac [52], as well as amide I in the vascular region [47] or amide II in the brain, heart, yolk sac, dorsum and tail of zebrafish larvae [39]. The width of this band had a high contribution to discriminate developmental stages at 96 and 120 hpf. The width of the band showed high values at 96 hpf and opposite low values of comparable magnitude at 120 hpf, which started to increase to average values thereafter. These changes may be indicative of differences in carotenoid molecules related to larvae developmental processes. In contrast, the frequency of band 3058 cm^−1^, usually associated with CH bonds, was very relevant at 24 hpf, tending to exhibit high values, then decreasing to average values thereafter.

In the heart, the most relevant Raman variables were F1639, W1639, A1639, A1375 and A1307 (Figure 6). Their vector module ranged from 0.45 to 0.51. Three of these variables were related with the same band (1639 cm^−1^; Figure 2). The three variables extracted from this band showed a similar pattern of variation across developmental stages, with lower values at 120 hpf and higher values at 144 hpf. The results suggest that strong variation occurs on the molecular conformations and their concentration and diversity at these developmental stages. In zebrafish, this band was previously associated with amide I in the brain and liver of adult animals [29,36], as well as with C=C bonds in the gonads of adult fish [29].

While many more bands/variables were obtained for the muscle tissue, compared to the remaining tissues and organs analysed, seven variables were found most relevant, some of them pertaining to the same band: F1199, F2852, W845, W1199, F845, A1199, and W796 (vector modules between 0.44 and 0.48; Figure 7). The bands from which these variables were derived have previously been associated with hydrocarbon bonds, aromatic amino acids, amides, nucleic acids and haemoglobin components (Figure 2). Furthermore, the region around the band 796 cm^−1^ has been related to O-P-O stretching, along with DNA in zebrafish larvae [15]. This location has been correlated with phosphodiester bridges and pyrimidine bases in the bone of the caudal fin of juveniles [14]. The DNA strand involves the connection of two nucleotides through a phosphodiester bond, with the phosphates (O-P-O bonds) linked to the nucleoside [57]. Band 1199 cm^−1^ was found to be allocated to amide III [29]. As noted, both amide I and amide III are usually related to the peptide backbone of proteins; their bands reflect coupled vibrations of the backbone, which depend on the secondary structure of the protein [38].

The most relevant bands found in the swim bladder were F1409, W1603, F1603, F1156, W1409, and W1002, with vector module ranging from 0.64 to 1.0 (Figure 8). These bands have mostly been related to amino acids and peptide bonds. Bands 1002 and 1603 cm^−1^ have been linked to the amino acids phenylalanine, tryptophan and tyrosine [36,39] (Figure 2). The variables clearly oscillated over development and were related to the diversity of molecular conformations present. Previous investigations in zebrafish have shown the swim bladder can be affected by exposure to different environmental contaminants, such as dioxin, cadmium (Cd), or eugenol [20,58,59]. The swim bladder is an organ filled with gas, which regulates buoyancy and motility. It is crucial for the survival of most teleost fish. In zebrafish, the swim bladder development starts during embryogenesis, and the inflation takes place after hatching. Alterations in the swim bladder and its development appear as relevant to detect and assess the impact of environmental contaminants. For example, a 1 h exposure of 4 hpf embryos to 1 µg/L dioxin was found to arrest the development of the swim bladder during its growth/elongation phase [58]. Exposure for 192 hpf to ≥250 µg Cd/L (up to 2 mg/L) also led to non-inflation of the swim bladder, associated with down-regulation of several genes of the Wnt and Hedgehog pathways, which regulate embryogenesis and cellular differentiation [59]. The metal altered the distribution of surfactant liquid and impaired the formation of the tissue layers (epithelial layer surrounded by a mesenchymal layer and then the outer mesothelium). These alterations were elicited via the production of reactive oxygen species, as they could be recovered by the addition of reduced glutathione to the medium [59]. Exposure for 96 h of 24 hpf embryos to 10–30 mg/L eugenol also reduced the inflation rate of the swim bladder, among other observed effects [20]. The effects probably occurred through inhibition of the Wnt/β-catenin signalling pathway regulating swim bladder development during the hatching and mouth-opening stages. Nevertheless, the methods employed in those studies take considerable time and resources. Herein, the swim bladder was very well characterised by the Raman spectra obtained. The bands and variables identified thus present themselves as of great interest to assess impaired development and function of the swim bladder elicited by toxicant exposure.

Overall, the work developed suggests a baseline profile for various organs and tissues of zebrafish embryos and larvae, identifying Raman bands of expeditious acquisition and their expected variation over different developmental stages, before and after hatching.

## 4. Conclusions

The work developed was based on in vivo RS acquisitions at early developmental stages of zebrafish representing a useful alternative to animal experimentation, within the Three Rs principle. A wealth of information was obtained, useful to characterise the developmental dynamics of relevant tissues and organs from 24 to 168 hpf. The RS bands identified represent valuable indications of molecules showing crucial changes over development and their wavenumbers. The results are relevant to plan future studies investigating responses to environmental cues, including for mechanistic purposes aiming at functional investigations of the organs and tissues under different exposure conditions. Future research should focus on investigating the effects of relevant chemical contaminants on zebrafish embryos and larvae with RS and investigate the utility of this technology within the risk assessment framework of adverse outcome pathways.

## Data Availability

Raw data and baseline corrected spectra available at Zenodo, https://zenodo.org/records/13937098.
